# Warming off southwestern Japan linked to distributional shifts of subtidal canopy-forming seaweeds

**DOI:** 10.1002/ece3.391

**Published:** 2012-10-12

**Authors:** Kouki Tanaka, Seiya Taino, Hiroko Haraguchi, Gabrielle Prendergast, Masanori Hiraoka

**Affiliations:** 1 Kuroshio Biological Research Foundation560 Nishidomari, Otsuki, Kochi, 788-0333, Japan; 2 Kochi Prefectural Fishery Experiment Station1153-23 Haigata, Uranouchi, Susaki, Kochi, 785-0167, Japan; 3 Coastal Branch of Tottori Prefectural Museum1794-4 Makidani, Iwami, Tottori, 681-0001, Japan; 4 Education Board of Shimanto City4-10 Ohashidouri, Nakamura, Shimanto, Kochi, 787-0033, Japan; 5 Usa Marine Biological Institute, Kochi University194 Inoshiri, Usa, Tosa, Kochi, 781-1164, Japan

**Keywords:** climate change, distributional shifts, *Ecklonia*, ENSO, Kuroshio Current, ocean warming, *Sargassum*, sea surface temperature, seaweeds

## Abstract

To assess distributional shifts of species in response to recent warming, historical distribution records are the most requisite information. The surface seawater temperature (SST) of Kochi Prefecture, southwestern Japan on the western North Pacific, has significantly risen, being warmed by the Kuroshio Current. Past distributional records of subtidal canopy-forming seaweeds (Laminariales and Fucales) exist at about 10-year intervals from the 1970s, along with detailed SST datasets at several sites along Kochi's >700 km coastline. In order to provide a clear picture of distributional shifts of coastal marine organisms in response to warming SST, we observed the present distribution of seaweeds and analyzed the SST datasets to estimate spatiotemporal SST trends in this coastal region. We present a large increase of 0.3°C/decade in the annual mean SST of this area over the past 40 years. Furthermore, a comparison of the previous and present distributions clearly showed the contraction of temperate species' distributional ranges and expansion of tropical species' distributional ranges in the seaweeds. Although the main temperate kelp *Ecklonia* (Laminariales) had expanded their distribution during periods of cooler SST, they subsequently declined as the SST warmed. Notably, the warmest SST of the 1997–98 El Niño Southern Oscillation event was the most likely cause of a widespread destruction of the kelp populations; no recovery was found even in the present survey at the formerly habitable sites where warm SSTs have been maintained. Temperate *Sargassum* spp. (Fucales) that dominated widely in the 1970s also declined in accordance with recent warming SSTs. In contrast, the tropical species, *S. ilicifolium*, has gradually expanded its distribution to become the most conspicuously dominant among the present observations. Thermal gradients, mainly driven by the warming Kuroshio Current, are presented as an explanation for the successive changes in both temperate and tropical species' distributions.

## Introduction

According to the Intergovernmental Panel on Climate Change, 4th report (IPCC 2007), the average Northern Hemisphere temperature during the second half of the 20th century was very likely higher than during any other 50-year period in the last 500 years, and the global climate has warmed by approximately 0.74°C in the last 100 years (1906–2005). Significant changes in biological systems are occurring on all continents and in most oceans due to global climate change (Rosenzweig et al. 2008). The most basic prediction of ecological responses to increasing temperatures is that species' distributions, influenced by the climatic regime, should shift toward the poles or higher altitudes (Walther et al. 2002; Harley et al. 2006). Following these predictions, distributional shifts of a wide range of terrestrial taxa have been well documented (Hickling et al. 2006; Parmesan 2006). There are overwhelmingly more publications of changes within terrestrial than marine ecosystems in response to climate change (Richardson and Poloczanska 2008; Hoegh-Guldberg and Bruno 2010; Wernberg et al. 2011a). Nevertheless, several shifts of marine organisms have been reported (e.g., Harley et al. 2006; Parmesan 2006; Hawkins et al. 2008; Sorte et al. 2010). However, available information on marine organisms' range shifts is relatively rare, because many researchers have indirectly assessed expansion or contraction of species' ranges using relative abundance of species at a single location as a proxy for spatial shifts (Rivadeneira and Fernández 2005; Harley et al. 2006). Moreover, a lack of adequate historical data prevents determination of whether distributional shifts in marine species to climate change have occurred or not (Merzouk and Jhonson 2011). Therefore, successive distributional records with a broader regional range and with decadal time scales are vital for assessing distributional shifts of marine species and their processes in response to recent warming.

**Figure 1:**
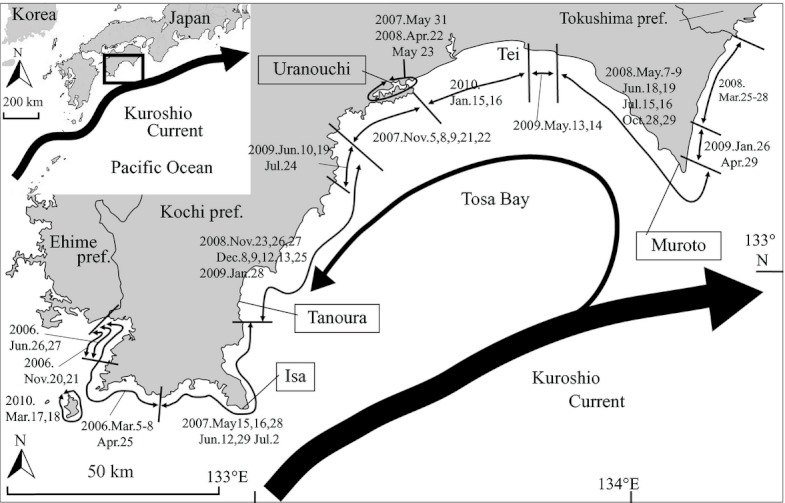
Map of observation region in Kochi, southwestern Japan. Thin arrows show observation periods. Thick and medium arrows show the main axis of the warm Kuroshio Current and the long-term dominant surface current inflow to Tosa Bay, respectively (Kuroda et al. 2008). Enclosed names show SST measurement locations.

In contrast with migratory species, range shifts in sedentary species occur via the slow processes of population extinctions and colonizations. This has made it easier to detect true geographic shifts because change is more methodical and missing data are less of an issue (Walther et al. 2002). Sedentary seaweeds serve as useful bioindicators that herald possible environmental changes to marine ecosystems (Tribollet and Vroom 2007). Canopy-forming seaweeds are the main component species on most temperate rocky shores, where two groups, kelps (Laminariales) and *Sargassum* (Fucales), are particularly abundant (Wernberg et al. 2011b). They play important roles as habitat providers, food source, and nursery for many other organisms, such as fish and shellfish, and thus changes in their distribution may affect a large number of species. However, relatively little is known about links between ocean warming and these key structural seaweeds (Lima et al. 2007; Merzouk and Jhonson 2011; Wernberg et al. 2011b; Díez et al. 2012). In the coastal region of Kochi facing the North Pacific Ocean in southwestern Japan, there are both historical records of seaweed distribution and long-term surface seawater temperature (SST) datasets. This offshore region has seen one of the most significant SST increases in the world, approximately 1.3°C over the last 100 years (Japan Meteorological Agency 2011). It has recently been reported that the coastal SST off the Northwest Pacific area has been warming up to 0.5°C per decade in the period between 1982 and 2010 (Lima and Wethey 2012) and that the SST warming rate over the path of the Kuroshio Current is two to three times faster than the global mean SST warming rate (Wu et al. 2012). The Kuroshio Current constantly carries warm tropical water to the Kochi region (Fig. 1), hence the Kochi coastline is also predicted to have warming SSTs. SST data have been recorded at several seaweed habitat sites along the Kochi coastline from the beginning of the 1970s. In addition, the distribution and abundance of dominant seaweed beds in the 1970s, 1980s, and 1990s has been recorded by Kochi Prefectural Fishery Experiment Station and Usa Marine Biological Institute, Kochi University (Kubota et al. 1979; Nature Conservation Bureau of Environment Agency of Japan, Marine Park center of Japan 1994; Ura 1999), because the importance of the seaweed beds to local fisheries has been well recognized in this region, where many fishermen rely on abalones and juvenile yellowtails, catches of which are strongly influenced by seaweed abundance. The abalone catch has been found to decrease with declining kelp populations (Kawajiri et al. 1981; Serisawa et al. 2004). Therefore, this region is well suited to studying the relationship between seaweed distributional processes at the decadal scale and warming SST trends in their habitat. Thus, we conducted a thorough survey of the major seaweed species' distributions in this area. We demonstrate a warming SST trend in this region and, by comparing our present observations to the previous distributional records, we report evidence of a successive shift of the distribution and population sizes of temperate and tropical seaweeds.

## Materials and Methods

### Analysis of long-term sea water temperatures

SST data recorded by Kochi Prefecture were analyzed in order to clarify long-term changes of SST for the whole investigation area. SST measurements were carried out at four locations (Fig. 1); three were open-shore environments, namely Muroto (east), Tanoura (west, within Tosa Bay), and Isa (west); one location was an inner-bay environment called Uranouchi (central, within Tosa Bay; Fig. 1). From 1970 to 2009, SST data were recorded at approximately daily frequency at each measurement location just offshore by drawing a bucket from the sea surface at the appointed hour.

The rates of long-term SST change at each location (ºC/decade) were calculated from annual mean SSTs recorded at each location, and the significance of each slope was calculated using regression analysis. Effects of time (data were split into eight 5-year periods from 1970 until 2009, inclusive) and location on SST were analyzed using GLM ANOVA (with fully factorial sums of squares) in SPSS 17. Data were separately assessed by season (April to June as spring, July to September as summer, October to December as autumn, and January to March as winter; with a Bonferroni corrected alpha value of 0.0125) and Tukey's post hoc tests revealed the sources of variation within the data.

The four SST measurement locations are differentially intercepted by the Kuroshio Current, which passes Isa and Muroto most directly (Fig. 1), Tanoura after its anticlockwise portion circulates, and finally Uranouchi, which being a sheltered area, should be the least influenced by this current.

### Previous distribution of Laminariales and Fucales

There are previous reports about the distribution of seaweed beds of >1 ha in this region from the 1970s (Kubota et al. 1979), 1980s (Nature Conservation Bureau of Environment Agency of Japan, Marine Park center of Japan 1994), and 1990s (Ura 1999). The reports showed the seaweed beds to be mainly composed of *Ecklonia* (Laminariales) and *Sargussum* (Fucales). In this study, the distributional maps of Laminariales and Fucales from the 1970s, 1980s, and 1990s were rearranged based on figures, tables, and field notes offered by the authors of these previous studies.

### Distributional survey

The official length of Kochi Prefecture's coastline (N 32°55′30.07″ E 132°39′18.74″–N 33°32′55.07″ E 134°18′20.54″), the study area, is 713 km (According to information published by Kochi Prefecture and a Japanese government Ministry). A total of 2,401 observation locations were set up every 200–300 m along the entire 713 km coast from March, 2006 to March, 2010 (Fig. 1). At each location, species of brown macro algae (Fucales and Laminariales) were recorded by snorkeling or using a glass-bottomed bucket. Observations were carried out around the time of low tide in order to see the 4–5 m depth bottom. The observation periods at several areas were consistent with those of previous research. However, because many Fucales population abundances vary seasonally in this region, some extra observations were made in different seasons for two limited southern and central areas, in order to avoid error by seasonal oversight.

Collected samples were identified with Yoshida (1998) and were taxonomically reconsidered with Mattio et al. (2009) and Mattio and Payri (2011) for *S. ilicifolium* and *S. aquifolium,* that had been identified as *S. duplicatum* and *S. crasifolium,* respectively, by Yoshida (1998). The distributional ranges of all recorded species of Laminariales encompassed only the temperate Japanese coast, thus all were categorized as temperate species. Among the Fucales, three types of species were categorized according to their distributional range. In general, *Sargassum* subgenus *Bactrophycus* is categorized as temperate (Yoshida 1983, 2004). However, even in the *Sargassum* subgenus *Bactrophycus*, *S. hemiphyllum, S. muticum*, *S. thumberugii,* and *S. fusiforme* are also distributed along tropical coastlines of the Western Pacific (Phillips 1995; Yoshida 1998), therefore these four species were categorized as having a wide range. The *Sargassum* subgenus *Sargassum* is generally distributed in tropical areas (Phillips 1995) and is categorized as a tropical species (Yoshida 2004), except for the wide range species *S. piluliferum* and *S. patens*.

To create a distributional map for recorded species, the positional coordinates recorded at all observation locations by handy GPS (Colorado300 GARMIN) were transformed to a positional map of each location using mapping software (Kasimir3D), and were applied to the contour maps using PC image software (Adobe Illustrator) along with information of any Fucales and Laminariales recorded at all observation locations. To estimate seaweed bed areas, those with up to 5% covered density of seaweed were marked on a contour map at the time of field observation. In addition, because *Ecklonia* (Laminariales) made beds at a depth of approximately 13m, extra observations were made by SCUBA diving along 12 transects in Tanoura, and bed areas were marked on a contour map. Marked seaweed bed areas on a contour map were measured by planimeter (PLANIX7 Tamaya Technics Inc.). Detailed analysis of the seaweed distribution will be reported separately. For the purposes of this study, data from the same location as the past reports were extracted to facilitate comparison and to reveal long-term trends.

## Results

### Long-term change in sea water temperatures

#### Annual mean SST trends

**Figure 2:**
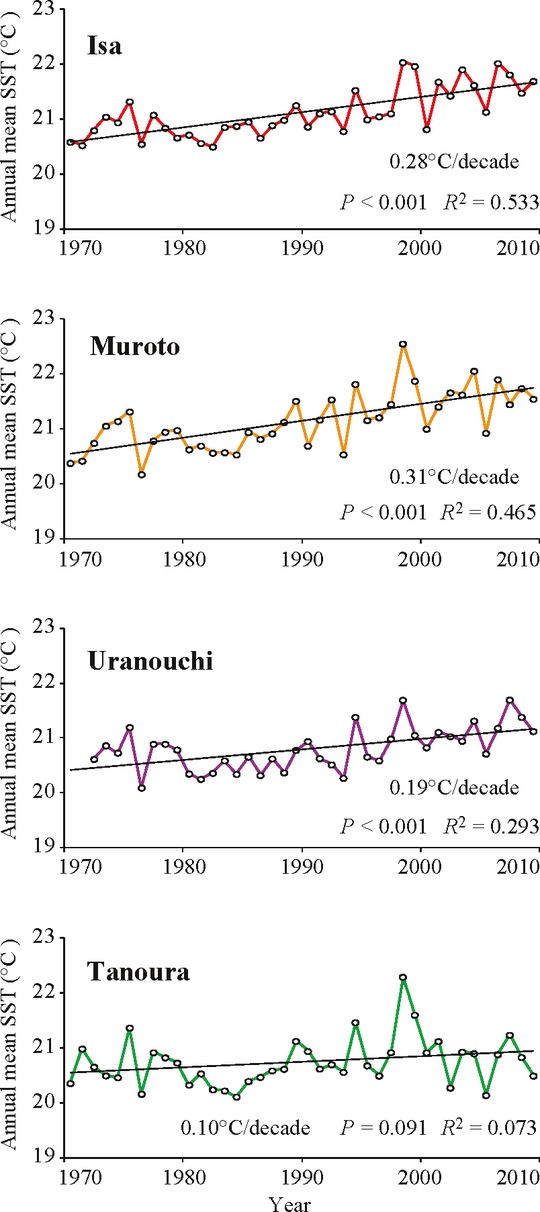
Annual mean SSTs recorded from 1970 to 2009 at four measurement locations in Kochi.

Annual mean SSTs recorded from 1970 to 2009 have warmed at all four measurement locations in the region (Fig. 2). Isa and Muroto, both located near the main axis of the Kuroshio Current, and Uranouchi, an inner-bay environment, underwent SST increases of 0.28 °C/decade (*r*^2^ = 0.533, *P* < 0.001), 0.31°C/decade (*r*^2^ = 0.465, *P* < 0.001), and 0.19°C/decade (*r*^2^= 0.293, *P* < 0.001), respectively. The gentle SST increase of 0.10°C/decade at Tanoura, located furthest from the Kuroshio Current, was not significant (*r*^2^ = 0.073, *P* = 0.091).

In terms of general decadal trends, SSTs in the 1980s were cooler than in the 1970s, but those of both the 1990s and 2000s were warmer than the 1970s (Fig. 2). The highest annual mean SST was recorded in 1998 among all measurement locations.

**Figure 3:**
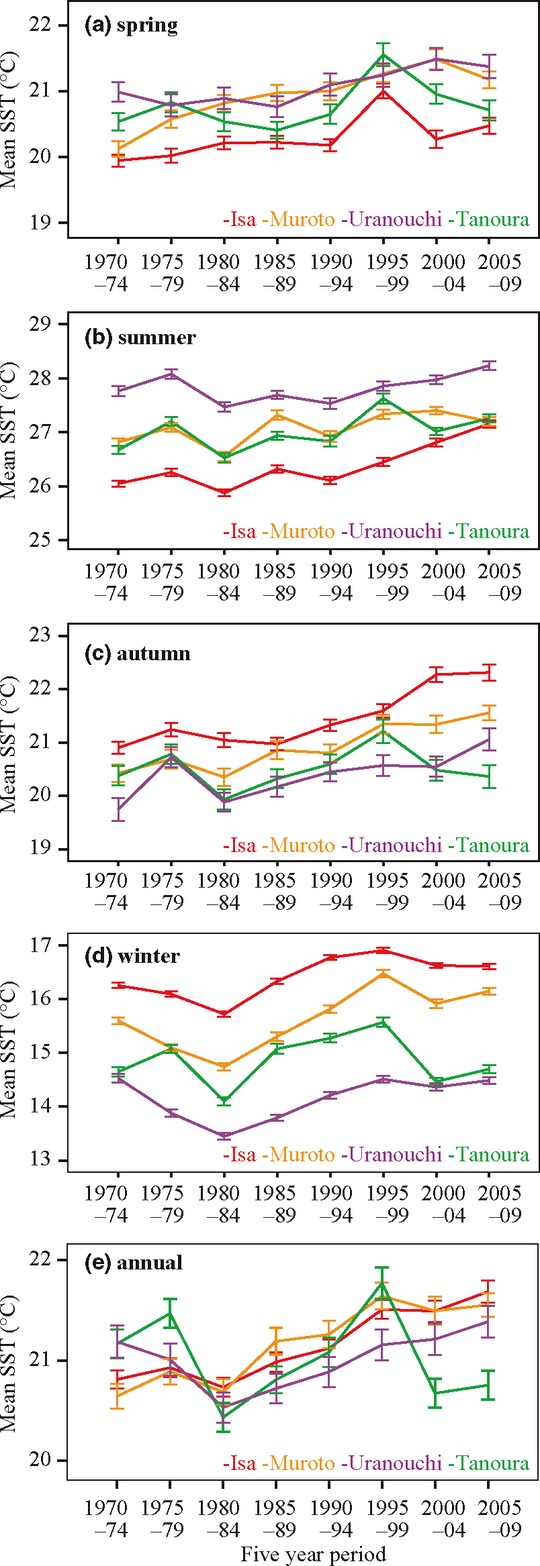
SST trends calculated for 5-year periods at four locations in each season [April to June as spring, (a); July to September as summer, (b); October to December as autumn, (c); and January to March as winter, (d)] and annual means (e). Error bars show ± 1SE.

There was a significant interactive effect of time (5-year period) and location on annual mean SST. At the beginning of the study (1970–74), Muroto and Isa, the two locations projecting into the Kuroshio Current, were similar (mean annual SSTs 20.64°C and 20.81°C, respectively; Fig. 3e). Furthermore, Isa was similar to Tanoura (21.17°C) and Uranouchi (21.19°C), yet Muroto was significantly cooler than Uranouchi and Tanoura (SS=255.083, MS= 85.028, df = 3, F = 4.287, *P* = 0.005). By the end of the study (2005–2009), this arrangement has changed in that three sites (Uranouchi 21.39°C, Muroto 21.55°C, and Isa 21.68°C) are now significantly warmer than Tanoura (20.75°C) (SS = 658.510, MS = 219.503, df = 3, F = 9.540, *P* < 0.001; Fig. 3e). As Uranouchi is sheltered from the path of the Kuroshio Current, however, it will be excluded from discussions of any changes in the Current's SST.

#### Seasonal SST trends

There were highly significant interactive effects of time (half decade) and location on SST during all seasons (spring, *n* = 10,944, MS = 16.357, df = 21, F = 2.614, *P* < 0.001, Fig. 3a; summer, *n* = 11,021, MS = 13.070, df = 21, F = 6.009, *P* < 0.001, Fig. 3b; autumn, *n* = 10,745, MS = 27.592, df = 21, F = 3.054, *P* < 0.001, Fig. 3c; and winter, *n* = 10,356, MS = 20.833, df = 21, F = 15.397, *P* < 0.001, Fig. 3d). Tukey's post hoc test revealed that overall, there was a highly significant (*P* < 0.001 in all cases) increase in SST between the start and the end of this study (half-decade periods 1970–74 and 2005–09) during spring, summer, and autumn by 0.59, 0.73, and 0.95°C, respectively (the slight increase in winter of 0.05°C was not significant), although these values mask the variations by location. In addition, as Uranouchi is an inner-bay environment, it is less exposed to the path of the Kuroshio Current and fluctuates greatly by season.

### Component species

**Figure 4:**
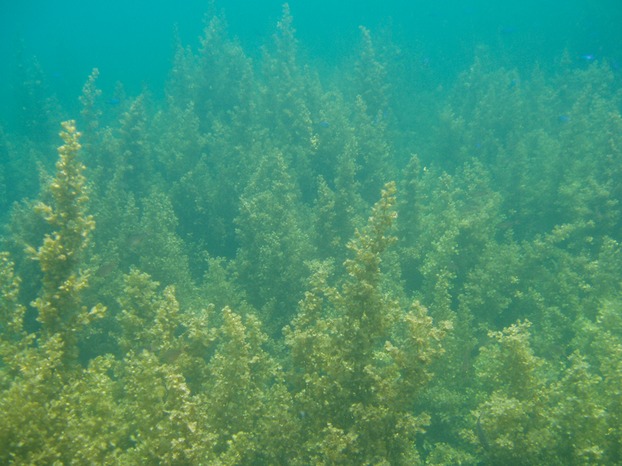
*Sargassum ilicifolium*. This tropical species is the most dominant, and has expanded its distributional ranges in the study area.

Table 1 shows the component species and the number of locations of brown seaweeds. Of 2,401 observation locations, brown seaweed appeared at 929 locations, Fucales and Laminariales appeared at 899 and 66 locations, respectively, and were absent at 1,472 locations. Therefore, brown seaweeds were recorded at 38.7% of locations; 37.4% and 2.7% for Fucales and Laminariales, respectively. Four Laminariales and 19 Fucales species were found in this study. The most commonly observed Laminariales were *Ecklonia cava* and *E. kurome*. The most commonly observed Fucales species were *Sargassum ilicifolium* (Fig. 4), and subsequently *S. okamurae S. micracanthum*. *Sargassum ilicifolium*, the most dominant species in the present region, is mainly distributed in the tropical Pacific and Taiwan (Yoshida 1998; Mattio et al. 2009), and belongs to the tropical *Sargassum* subgenus. Other species that belong to the tropical *Sargassum* subgenus were also recorded. Temperate *Bactrophycus* subgenus species, *S. okamurae* and *S. micracanthum* were also frequently recorded. Although most species were mainly distributed along exposed shore environments, rarely, species requiring sheltered conditions were also found in the inner-bay environment, notably, tropical *S. carpophyllum* and *S. aquifolium*.

**Table 1:** Component species of observed canopy-forming seaweeds. The location column shows the number of locations where a given species, order or no seaweed was recorded, as well as the total of observation locations. See text about categorizing species as tropical or temperate types

	Subgenenus	Species	Tropical	Temperate	Wide range	Location	Appearance ratio (appearance locations/total locations)
Fucales	*Sargassum*	*Sargassum ilicifolium*	○			271	11.3%
		*S. piluliferum*			○	73	3.0%
		*S. alternato-pinnatum*	○			34	1.4%
		*S. carpophyllum*	○			16	0.7%
		*S. crispifolium*	○			11	0.5%
		*S. patens*			○	6	0.2%
		*S. assimile*	○			5	0.2%
		*S. aquifolium*	○			2	0.1%
		*S. kushimotense*	○			1	0.0%
	*Bactrophycus*	*S. okamurae*		○		270	11.2%
		*S. micracanthum*		○		179	7.5%
		*S. nipponicum*		○		81	3.4%
		*S. hemiphyllum*			○	73	3.0%
		*S. yamamotoi*		○		52	2.2%
		*S. fusiforme*			○	16	0.7%
		*S. tenuifolium*		○		6	0.2%
		*S. thunbergii*			○	5	0.2%
		*S. muticum*			○	5	0.2%
	?	*S. sp*.		○		4	0.2%
Laminariales		*Ecklonia cava*		○		29	1.2%
		*E. kurome*		○		20	0.8%
		*Eckloniopsis radicosa*		○		3	0.1%
		*Undaria undarioides*		○		19	0.8%
Total Fucales						899	37.4%
Total Laminariales						66	2.7%
Total canopy-forming seaweeds						929	38.7%
*No* canopy-forming seaweeds						1472	61.3%
Total observation locations						2401	

### Distributional change in Laminariales

**Figure 5:**
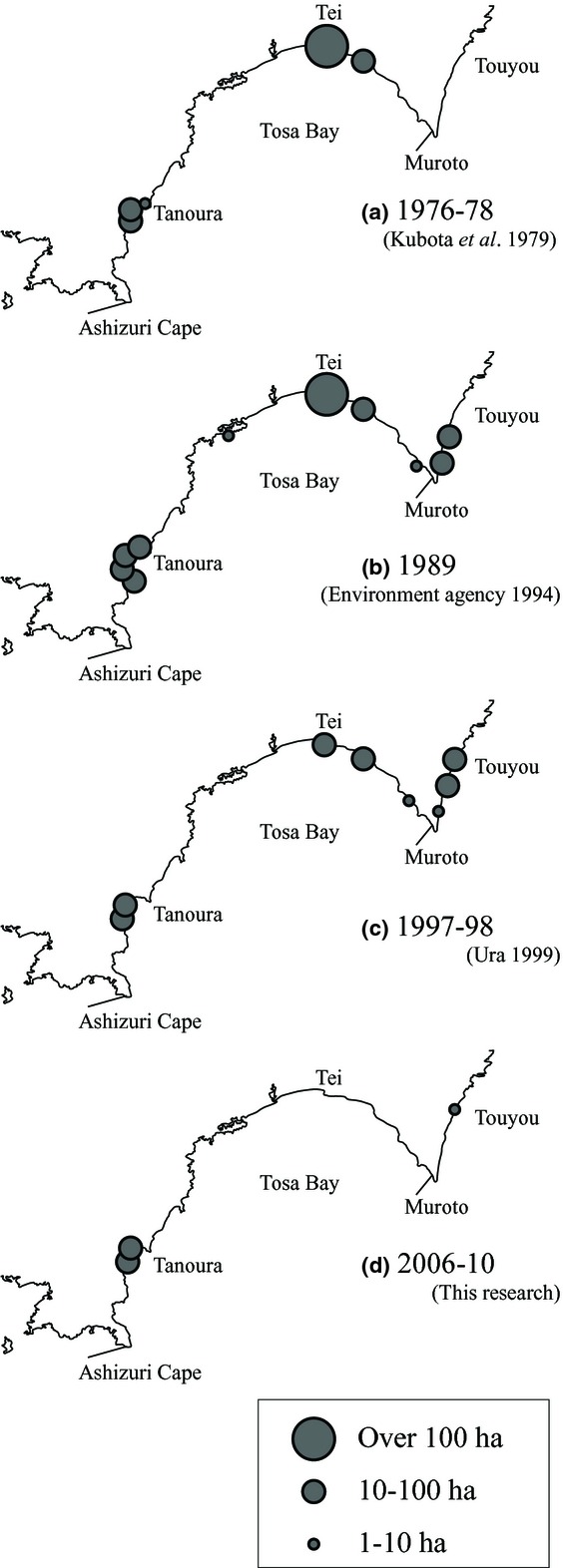
Distributional shifts of temperate *Ecklonia* populations. Distribution of 1970s, 1980s, 1990s and 2000s are shown in (a), (b), (c) and (d) respectively. Environment agency (1994) is the report of the Nature Conservation Bureau of the Environment Agency of Japan and the Marine Park center of Japan.

Fig. 5a–d summarizes the distribution and relative population size of the main kelp, the temperate *Ecklonia* genus, in the Kochi region, southwestern Japan, since about 30 years ago. In the previous reports (Migita 1984), *E. cava* and *E. kurome* were not distinguished because both species have similar morphology and can hybridize each other. Therefore, the two species are also not distinguished in the present distributional map. According to previous reports in this region, the temperate *Ecklonia* populations in several areas expanded their distribution and each population underwent large scale increases from the 1970s to the 1980s (Fig. 5a–b). In particular, the largest population in Tei (central, within Tosa Bay) grew up from 100 to 180 ha in this period. However, the exuberance of *Ecklonia* decreased in the 1990s (Fig. 5c); moreover, the number of populations declined to only two (Tanoura and Touyou) remaining from 2000s onward (Fig. 5d).

### Distributional change in Fucales

**Figure 6:**
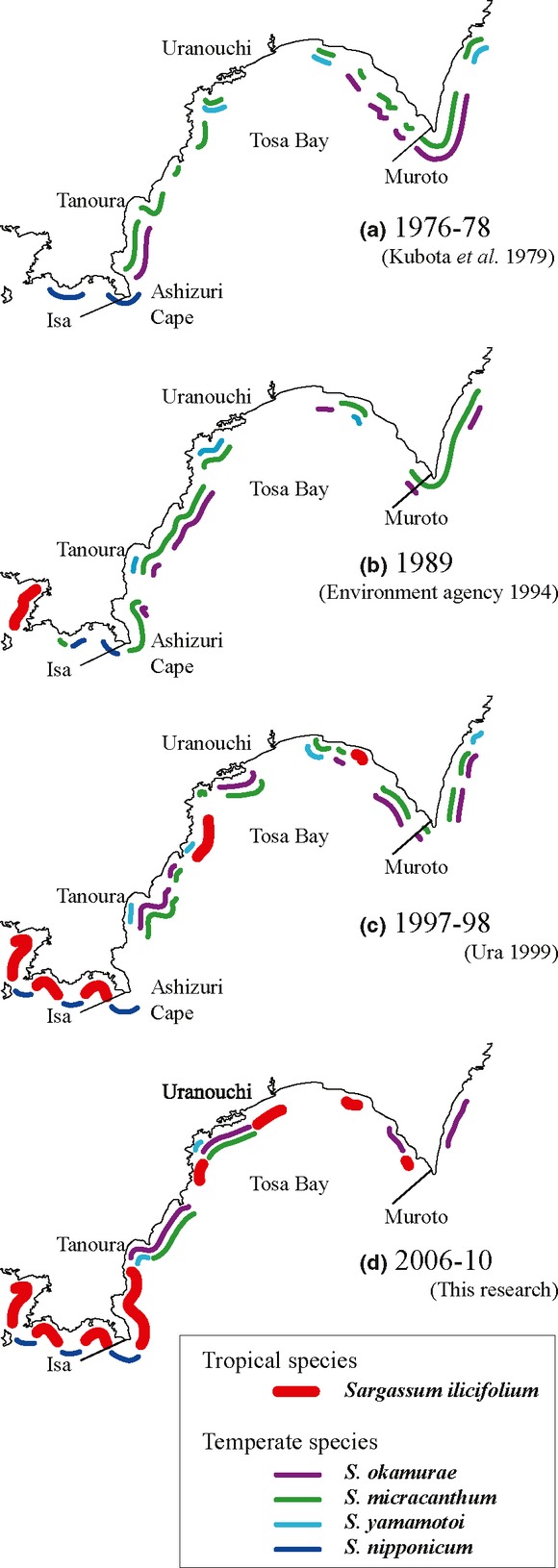
Distributional shifts of main *Sargassum* spp. populations. Distribution of 1970s, 1980s, 1990s and 2000s are shown in (a), (b), (c) and (d) respectively. Environment agency (1994) is the report of the Nature Conservation Bureau of the Environment Agency of Japan and the Marine Park center of Japan.

Fig. 6a–d summarizes the distribution of the five most common *Sargassum* spp. populations. In the 1970s, all *Sargassum* beds were composed entirely of temperate species (Fig. 6a). In the late 1980s, tropical *S. ilicifolium* populations initially appeared in the most western locations, while the temperate populations of *S. okamurae* in the eastern locations around Muroto Cape declined (Fig. 6b). From the 1980s to the 2000s, tropical *S. ilicifolium* further expanded its distribution in Tosa Bay, becoming the most common species in this region, while the three temperate species *S. okamurae*, *S. micracanthum,* and *S. yamamotoi* further declined (Fig. 6c,d). Most notably, the temperate populations of *S. micracanthum* and *S. yamamotoi* disappeared from the eastern locations around Muroto Cape. However, temperate populations of *S. nipponicum* dominant in the southern locations did not change their range.

## Discussion

### Laminariales

It has long been known that temperature controls kelp distribution and abundance. On the eastern coast of the North Pacific, the giant kelp, *Macrocystis pyrifera* forest decreased during a period of warming SST and then recovered during a subsequent SST cooling (Edwards 2004; Ladah and Zertuche-González 2004). In central Japan's Shizuoka, on the western coast of the North Pacific, a severe decline of temperate *Ecklonia* populations correlated with warming SST in the coastal area with the approach of the Kuroshio Current alongshore (Kawajiri et al. 1981). In this study, expanding population distributions occurred during the cooling period from the 1970s to the 1980s, while population decline occurred in the warmer periods from the 1980s to the 2000s (Fig. 2 and 5). Such *Ecklonia* population fluctuations in response to changing SST are consistent with the reports from other regions. In particular, the kelp decline during the remarkably high SSTs at the end of the 1990s bears a striking correspondence to the eastern region of the North Pacific Ocean. Serisawa et al. (2000, 2004) recorded the decline process of the largest *Ecklonia* population in Tei in this period, from 1991 to 2001. According to their reports, *Ecklonia* plants with damaged blades were found in 1997; plant growth was particularly poor in 1999 and had completely disappeared by 2000. The decline of the giant kelp forest in the eastern North Pacific also occurred within this period of time (1997–98; Edwards 2004; Ladah et al. 1999). In addition, severe coral bleaching was reported from many coral reefs in the tropical and subtropical regions of the world during 1997–98 (Wilkinson 1998), including in southern Japan (Kayanne et al. 2002). The El Niño Southern Oscillation (ENSO) of 1997–98 was the strongest on record with major climatic impacts felt around the world (McPhaden 1999a,b; Overland et al. 2001; McPhaden et al. 2006). The reported negative impacts to kelps and corals in 1997–98 have been attributed to the high SSTs brought about by ENSO (Ladah et al. 1999; Kayanne et al. 2002; Edwards 2004). Annual mean SSTs in 1998 at all sites in our region were remarkably high (Fig. 2). In this context, the destruction of the largest *Ecklonia* population in the present region of the western North Pacific may be attributed to the high-magnitude ENSO event.

In the eastern North Pacific, recoveries of kelp populations after the warm SSTs of 1997–98 were found when the SST cooled again. The recovery returned local control of giant kelp populations within 6 months in southern California, and within 2 years in Baja California, by the rapid establishment of a cold period (1998–1999 La Niña; Edwards 2004). However, in our study region, recovery of *Ecklonia* populations has not been found more than 10 years after the 1997–98 ENSO. As a result of not only the temporarily warmer SST event caused by ENSO but also of the persistently warmer SSTs that followed (Fig. 2 and 3), *Ecklonia* populations apparently have not recovered from around Muroto Cape to Tei, the eastern inside of Tosa Bay (Fig. 5).

High temperature regimes are known to negatively influence the physiological responses of *Ecklonia*. Net photosynthesis of the *Ecklonia* fronds has a negative value under low light intensity of <12.5 μmol/m^2^/s and high temperatures >25°C (Kurashima et al. 1996). *Ecklonia* juveniles recruit and grow under a canopy of their adult plants during autumn to winter (Serisawa et al. 2001). In Muroto, a significant SST warming was found during all seasons, in particular during autumn (+1.1°C over 40 years; Fig. 3). Thus, the decline of *Ecklonia* populations around Muroto may have resulted from the autumn SST warming having negatively influenced juvenile growth conditions under the adult canopy. On the other hand, Tanoura's *Ecklonia* populations may have persisted because the local SST cooled again after the warmest period of 1995–1999 (Fig. 3).

Warming SST can not only directly affect the physiological responses of *Ecklonia* but also indirectly, such as via effects on herbivory. Feeding by herbivorous fishes has been identified as one of the potential causes of seaweed bed decline, and herbivorous damage to *Ecklonia* populations has been reported in several regions of Japan (Nakayama and Arai 1999; Masuda et al. 2000). The rabbit fish, *Siganus fuscescens*, and the parrot fish, *Calotomus japonicus*, as phytophagous fish of *Ecklonia*, have optimal temperatures for eating at over 20°C and around 18°C, respectively (Kimura 1994). In fact, in western Japan, it has been demonstrated that the recent SST warming in winter has extended the period of herbivorous fish activity by at least 2 months (Yamaguchi et al. 2010). Regarding the disappearance process of the largest *Ecklonia* population in this study, the damaged bladelets and blades observed in many plants is inferred to be due to grazing by fishes (Serisawa et al. 2004). It can be presumed that the warming SST is extending the eating behavior of these herbivorous animals on *Ecklonia* populations. A further study of the impact of various herbivores on *Ecklonia* under varying SST conditions should be conducted.

### Fucales

For *Sargassum* spp., the expansion of tropical and contraction of temperate species' distributional ranges were clear in this region (Fig. 6). *Sargassum ilicifolium,* which has most conspicuously increased in this study, is mainly distributed in tropical countries, and southern Japan is at the northern edge of its range (Mattio et al. 2009). As for the obvious decline of the three main temperate species, *S. okamurae*, *S. micracanthum,* and *S. yamamotoi*, Kochi coastlines are almost at their southern margin (Yoshida 1983). Although the other temperate species, *S. nipponicum,* is also nearly at its southern margin and yet shows a stable long-term distribution, samples have been collected at a location more than 100 km southward at the neighboring island of Kyushu (Yoshida 1983). Therefore, *S. nipponicum* may not reach their absolute range limits in this region. There is less evidence that northern species are retreating at their lower latitudinal margins in comparison with the distributional expansions of many southern species seen at their higher latitudinal margins. It has been pointed out that recording or historical information with imprecise spatial scales may make it difficult to identify declines at lower latitudinal margins (Wilson et al. 2005). However, as we obtained ample historical records of temperate species' distributions, perhaps the current research indeed demonstrates a successive decline process from the southern margins of these temperate species' ranges.

Most of Kochi's coastline is exposed shore and inner-bay conditions are limited. However, some tropical species preferentially inhabiting inner-bay environments have also expanded. Although a small population of tropical *S. carpophyllum* was previously reported in limited bays (Noro 2004; Haraguchi et al. 2006; Kobayashi 2008), we found more populations, which were also larger, and tropical *S. aquifolium* was found for the first time. These inner-bay types of tropical species made mixed communities with temperate species such as *S. hemiphyllum*, *S. yamamotoi,* and *S. piluliferum*. As Uranouchi is a sheltered water body with limited mixing of ocean water inflow, its SST is more influenced by the air temperature, which has a large seasonal amplitude (Fig. 3b, d), but significantly increases in annual mean (Fig. 2). Thus, the expansion of tropical species in inner bays also seems to be synchronized to SST warming, even in inner-bay conditions.

### Influence of the Kuroshio Current

On the contraction processes of the temperate kelp, *Ecklonia* and the main three *Sargassum* spp.' distributional ranges, their populations declined at higher latitudes of the eastern side in Tosa Bay around Muroto Cape and persisted at lower latitudes from Tanoura to Uranouchi in the western side of Tosa Bay (Fig. 5d, 6d). On the expansion processes of tropical species, *S. ilicifolium* populations within Tosa Bay initially colonized in a disperse manner, also at the eastern side (Fig. 6c), and later expanded to lower latitudes (Fig. 6d). These contraction and expansion processes are explained by the thermal gradients formed by the warm Kuroshio Current, rather than by simple latitudinal gradients. As long-term (1950–2004) mean surface current patterns related to the Kuroshio Current generate an anticlockwise circulation in Tosa Bay (Kuroda et al. 2008; Fig. 1), SST isotherms should be the lowest in the western side of Tosa Bay with the longer path of the warm inflow and be higher in both the northern and southern ends of Tosa Bay directly struck by the Kuroshio Current. The predicted thermal pattern is supported by actual recorded SSTs at Tanoura, Isa, and Muroto. The annual mean SST is warmer at both bay ends, Isa and Muroto, which are also significantly warmer than at Tanoura, at the western interior of the bay (Fig. 3e). Warmer SST values at the bay ends agree with the accelerated warming over the path of the Kuroshio Current recently reported (Wu et al. 2012). Thus, we suggest that the warming of the Kuroshio Current and its positional fluctuations have essentially driven the expansion of tropical species' distributional ranges and the contraction of temperate species' distributional ranges in this region.

A high-resolution climate model under global warming has predicted an increase in the Kuroshio Current velocity, which will further accelerate northward heat transport (Sakamoto et al. 2005). A simulation-based study predicts that in the Atlantic Ocean, range shifts of phytobenthos will be affected by future SST warming (Müller et al. 2009). In this study, it is probable that if recent SST warming continues into the future, contraction of temperate species' ranges and expansion of tropical species' ranges in this region will continue, ultimately leading to an ecological transformation of the coastal ecosystems. The rapid shifts observed in this region are provided as a precedent for the impact of global warming to marine ecosystems. Moreover, seaweeds have accompanied human economic activity (Buchholz et al. 2012). In this study area, the decline of temperate kelp populations has caused the decline of abalone catches and their important associated income (Serisawa et al. 2004). Recently, changes of fish communities resulting from changing seaweed communities have also been described in this area (Terazono et al. 2012). It is likely that the changing marine community in response to the changes in seaweed populations and ranges will significantly impact local human economic activity into the future.
